# THE EFFECT OF LANGUAGE ON PREPARATION FOR OLD AGE: EVIDENCE FROM CROSS-CULTURAL SURVEY AND TWITTER DATA

**DOI:** 10.1093/geroni/igz038.3536

**Published:** 2019-11-08

**Authors:** Amber Xuqian Chen, Helene H Fung

**Affiliations:** The Chinese University of Hong Kong, Hong Kong, Hong Kong

## Abstract

We aimed to further investigate the linguistic-savings hypothesis (Chen, 2013) in the field of aging, which maintains that when languages grammatically divide the future and the present (e.g. English and Czech), speakers tend to behave less future-oriented than those speaking languages that do not mark future tense (e.g. German and Chinese). In the 2018 wave of Aging as Future Project, 2,042 participants from the United States, Germany, Czech Republic, Hong Kong and Taiwan (18-93 year, Mean age= 55.47, 55.61% female) completed online questionnaires. The results supported the hypothesis that people speaking future-less languages tended to perceive the timing of preparation for old age closer to the present in terms of finance, living arrangement, nursing care, and loneliness, they also took action earlier and performed more relevant activities. Furthermore, the association between language and preparation timing was more salient in older adults than younger counterparts. And path analysis revealed that time discounting was a significant predictor (P=0.049) for the future-oriented behavior. Hence, speakers of futureless languages will view the future as temporally closer to the present, causing them to discount the future less and prepare for old age actively. Using LIWC 2017, we then analyzed community-level of future orientation with 80 million Tweets across countries and replicated our principal result through that usage of future-oriented languages partly predicted prevalence of health behaviors. The findings indicate that language not only shape people's own future-oriented outcomes, through decreasing time discounting, but also influence population health as a whole.

